# Pharmacogenomics in clinical practice: Biomarker information in Brazilian drug labels

**DOI:** 10.1002/bcp.70278

**Published:** 2025-09-28

**Authors:** Guilherme Suarez‐Kurtz

**Affiliations:** ^1^ Division of Clinical Research and Technological Development National Cancer Institute Rio de Janeiro Brazil

**Keywords:** biomarkers, clinical pharmacology, drug analysis, drug regulation, genetics and pharmacogenetics, pharmacogenomics

## Abstract

This review examines the PGx annotations in package inserts (*bulas* in Brazilian Portuguese) approved by ANVISA, the Brazilian Health Regulatory Agency, for 19 gene–drug pairs with strong or moderate recommendations for initial dosing alteration in the CPIC (Clinical Pharmacogenetic Implementation Consortion) guidelines and PGx testing required or recommended by health regulatory agencies listed in the PharmGKB Drug Label Annotations table. It is assumed that drug–gene pairs with these two features should be prioritized for adoption by the Brazilian Public Health System (SUS). PGx annotations were distributed across seven of the ten sections of the *bulas* and classified as PGx testing required (*n* = 5), PGx testing recommended (*n* = 5), actionable PGx (*n* = 4) and no PGx clinical information (*n* = 4). Pairwise comparison of assigned PGx levels in ANVISA *bulas vs*. the selected regulatory agencies revealed poor concordance (Cohen's kappa coefficient *κ* < 0.20 for all pairs); however, discordance among these agencies is also considerable (Fleiss's kappa coefficient *κ* = 0.08). The frequency of the examined PGx risk biomarkers in representative Brazilian cohorts range from <0.1% to 10.8%. Importantly, Native Americans (0.4% of the current Brazilian population), display wide PGx diversity, both interethnically and in relation to non‐Indigenous Brazilians. The author suggests that addition of a PGx section to the ANVISA *bulas* would avoid dispersion of clinically relevant PGx information across sections, assure integration of this information with SUS determinations and prevent discrepancies across sections, as observed in the *bulas* for thiopurines.

## 1 INTRODUCTION

The vast majority of individuals from distinct populations worldwide have at least one clinicaly “actionable” pharmacogenetic variant,^1^, ^2^, ^3^ which has been defined as “a variant that if present in the right gene combination … prescribing decisions would be altered from the standard course of action”.^4^ Thus, it appears intuitive that genetically guided drug prescription would be more effective and less toxic than the prevalent strategy of “one size fits all”; indeed, the influence of genetic predisposition on drug response is a tenet of precision medicine. Evidence‐based guidelines, such as those published by the Dutch Pharmacogenetics Working Group (DPWG)^5^ and the Clinical Pharmacogenetics Implementation Consortium (CPIC),^6^ facilitate the translation of PGx findings to prescribing decisions in clinical practice. Drug package inserts (drug labels, summary of product characteristics, etc.) represent additional sources of PGx information for prescribers. The Pharmacogenomics Knowledgebase (PharmGKB) provides a compilation of pharmacogenomic (PGx) information from five national drug regulatory agencies, that are classified according to the potential for clinical action as PGx testing required, PGx testing recommended, actionable PGx, informative PGx and no clinical PGx (https://www.pharmgkb.org/labelAnnotations).

Several studies have compared PGx information on package inserts for drugs marketed in different countries.^6^, ^7^, ^8^, ^9^, ^10^, ^11^ The present study extends this analysis to Brazil, using the PGx annotations in packge inserts (*bulas* in Brazilian Portuguese) approved by ANVISA, the Brazilian Health Regulatory Agency. Rather than attempting to cover all drugs marketed in Brazil, the author focused on gene–drug pairs with CPIC strong or moderate recommendations for initial dosing alteration and PGx test required or recommended by at least one of the regulatory agencies listed in the PharmGKB Drug Label Annotations table, namely FDA (Food and Drug Administration, US), EMA (European Medicines Agency, European Union), CHSC (Health Canada Santé Canada), Swissmedic (Swiss Agency for Therapeutic Products) and PMDA (Pharmaceutical and Medical Devices Agency, Japan). These inclusion criteria were established on the assumption that drug–gene pairs with these two features should be prioritized for adoption by the Brazilian Unified Health System (*Sistema Único de Saúde*, SUS). Indeed, at the present time, the three drug–gene pairs with PGx testing required, and provided by SUS, namely HLA‐B*57:01–abacavir, *CFTR*–ivacaftor and tafenoquine‐G6PD fulfil these inclusion criteria.

The distribution of PGx annotations across the various sections of the ANVISA *bulas* is examined, the ensuing PGx testing recommendations are classified and compared to those in the PharmGKB Drug Label Annotations Table, and the prevalence of the target biomarkers in representative cohorts is leveraged to explore the implications of PGx testing in Brazilians.

## 2 METHODS

The CPIC guidelines were accessed at https://cpicpgx.org/guidelines/ (links in Table S1), CPIC levels (A–D) and PharmGKB levels of evidence (1A–4) were obtained from https://cpicpgx.org/genes-drugs/. PGx information in drug labels approved by the regulatory agencies FDA, EMA, CHSC, Swissmedic and PMDA were extracted from the PharmGKB Drug Label Annotations table (https://www.pharmgkb.org/labelAnnotations). PGx information in ANVISA approved drug labels (“*bula do profissional*”) were derived from https://consultas.anvisa.gov.br/#/bulario/. The links to the most recent version of each *bula* referred to in the text are listed in Table S2. For comparison with other drug regulatory agencies, the PGx annotations in the ANVISA *bulas* were classified by the author according to the PGx level of actionability defined in PharmGKB, namely PGx testing required, PGx testing recommended, actionable PGx, informative PGx or no PGx clinical information (https://www.pharmgkb.org/page/drugLabelLegend#pgx-level). Of note, ANVISA annotations that PGx testing “should be *performed*” (e.g., CFTR risk variant for ivacaftor) were classified as “PGx testing required”, whereas annotations that PGx testing “should or must be *considered*” (e.g., HLA‐B*57:01 for abacavir) were classified as “PGx testing recommended”.

The prevalence in Brazilians of the PGx biomarkers included in this study were derived from: (i) the REDOME database of HLA alleles from 298 000 unrelated haematopoietic stem cell volunteer donors registered with the Brazilian Bone Marrow Donor Registry^12^; (ii) the Refargen database (www.refargen.org.br) comprising 1034 healthy adults, recruited at the four most populated regions of Brazil and self‐identified as White, Brown (*Pardo* ín Brazilian Portuguese) or Black, according to the terminology adopted by the Brazilian census; collectively, these three race/Colour categories in the four regions account for >90% of the overall Brazilian population (https://www.ibge.gov.br/en/statistics/social/labor/22836-2022-census-3.html?langZenGB). The term Colour and the Colour categories are capitalized to call attention to their meaning in the context of the Brazilian census: Colour (‘*cor*’ in Portuguese) denotes the Brazilian equivalent of the English term ‘race’. (iii) the SABE Project database, a high‐coverage whole‐genome sequencing data set of 1171 elderly individuals recruited in São Paulo, the largest metropole in Brazil (https://abraom.ib.usp.br/); (iv) two sets of DNA samples genotyped in the author's laboratory at the Brazilian National Cancer Institute (INCA), one set from 430 acute lymphoblastic leukaemia (ALL) paediatric patients and the other from 300 adults patients diagnosed with gastrointestinal tumours; (v) Pubmed, using as search terms the target gene and drug plus “Brazil*” (e.g. 6GPD AND primaquine AND Brazil*).

### 2.1 Statistical analysis

Kappa statistics were used to quantify inter‐agency agreement (concordance) on categorical PGx classification: Cohen's kappa coefficient was applied for pairwise comparisons and Fleiss's kappa coefficient for comparisons among >2 agencies. Calculations were made using excel templates available at https://real-statistics.com/. The kappa values were interpreted according to Landis and Koch^13^: *κ* < 0.20 indicates slight (poor, minimal) agreement; *κ* = 0.21–0.40 fair agreement; *κ* = 0.41–0.60 moderate agreement; *κ* = 0.61–0.80 substantial agreement; and *κ* = 0.81–1.0 almost perfect agreement.

## 3 RESULTS

**FIGURE 1:**
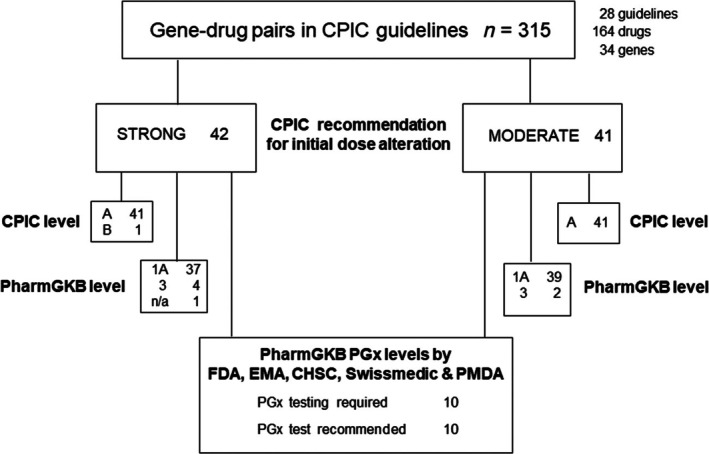
Flowchart of the search methodology and inclusion criteria.

Figure 1 presents a flowchart of the search results and Figure S1 shows a colour code plot of the dosing recommendations in the 28 CPIC guidelines analysed in this study. These guidelines cover 34 genes and 164 drugs, distributed in 315 unique gene–drug pairs; strong, moderate, optional or no recommendations for initial dosing apply to 128, 86, 37 and 15 gene–drug pairs, respectively, when aminoglycosides, volatile anaesthetics and high‐risk drugs metabolized by G6PD are analysed as single groups of drugs. “Strong” and “moderate” CPIC recommendations for change of initial dose or choice of alternate drugs (denoted together as “initial dosing alteration” hereafter) apply to 42 and 41 gene–drug pairs, respectively; the vast majority of these pairs are at CPIC level A (97.8%) and PharmGKB level of evidence 1A (91.6%). However, only 20 gene–drug pairs with strong and moderate CPIC recommendations for initial dosing alteration have PGx testing required or recommended by at least one of the regulatory agencies FDA, EMA, CHSC and Swissmedic, according to https://www.pharmgkb.org/labelAnnotations; of note, PMDA did not require or recommend PGx testing for any of these pairs, and consequently was not further considered.

The following sections deal with each gene–drug pair with respect to the PGx annotations in ANVISA‐approved *bulas* compared to the FDA, EMA, CHSC and Swissmedic drug labels, and the frequency of the target biomarkers in Brazilians. Table 1 summarizes these data.

**TABLE 1:** Assigned PGx levels for selected gene–drug pairs.

Biomarker				Ref.	Assigned PGx level^b^				PGx level^c^
	Risk genotype or phenotype (frequency, %)^a^				FDA	EMA	CHSC	SWISSMEDIC	ANVISA
HLA‐B*57:01	**Positive (3.7)**			12					
Abacavir	STRONG				REQUIRED	REQUIRED	REQUIRED	REQUIRED	Recommended
HLA‐B*15:02	**Positive (0.06)**			12					
Carbamazepine	STRONG				REQUIRED		Recommended	REQUIRED	Recommended
Oxcarbazepine	STRONG				Recommended		Recommended	REQUIRED	Recommended
Phenytoin	STRONG				Actionable		Recommended	Actionable	No clinical PGx
HLA‐A*31:01	**Positive (9.3)**			12					
Carbamazepine	STRONG				Actionable		Recommended	Recommended	Recommended
***CFTR risk variants***	**Carrier (n/a)**			n/a					
Ivacaftor	STRONG				REQUIRED	REQUIRED	REQUIRED	_	REQUIRED
**6GPD phenotype**	**Deficient (4.8)**			18					
Pegloticase	STRONG				REQUIRED	REQUIRED		_	Not marketed in Brazil
Primaquine	STRONG				REQUIRED		REQUIRED		Recommended
Rasburicase	STRONG				REQUIRED	Actionable	REQUIRED	Actionable	No clinical PGx
Tafenoquine	STRONG				REQUIRED			_	REQUIRED
**DPD phenotype**	**IM AS 1.5 (3.1)**	**IM AS 1.0 (0.4)**	**PM (0.4)**	19					
5‐fluorouracil	Moderate	STRONG	STRONG		Recommended		Recommended	REQUIRED	No clinical PGx
Capecitabine	Moderate	STRONG	STRONG		Recommended	Recommended	Recommended	Actionable	Recommended
**NUDT15 phenotype**	**IM (2.8)**	**PM (n/a)**		20					
Azathioprine	STRONG	STRONG			Recommended		REQUIRED		REQUIRED^d^
Mercaptopurine	STRONG	STRONG			Recommended	Recommended	Recommended	Actionable	REQUIRED^d^
Thioguanine	Moderate	STRONG			Recommended		Recommended		REQUIRED^d^
**TPMT phenotype**	**IM (9.8)**	**PM (1.0)**		20					
Azathioprine	STRONG	STRONG			Recommended		Actionable	Actionable	Actionable
Mercaptopurine	STRONG	STRONG			Recommended	Recommended	Recommended	Actionable	Actionable
Thioguanine	Moderate	STRONG			Recommended		Recommended	Actionable	Actionable
HLA‐B*58:01	**Positive (4.1)**			12					
Allopurinol	STRONG				Recommended		Recommended	Actionable	Actionable
**SLCO1B1 function**	**1**	**Poor (2.5)**		22					
Simvastatin		STRONG						Recommended	No clinical PGx

a Frequency in Brazilians.

b From the PharmGKB Drug Label Annotations table (https://www.pharmgkb.org/labelAnnotations).

c PGx testing classified by the author.

d Classified as REQUIRED based on section 3 of the *bula*, whereas the annotations in section 5 classify as PGx testing recommended.

### 3.1 HLA‐B*57:01– abacavir

FDA, EMA, CHSC and Swissmedic require PGx testing prior to administration of abacavir. Accordingly, the Brazilian Ministry of Health requires testing for HLA‐B*57:01 prior to inclusion of abacavir in antiretroviral combined therapy for HIV infection; the test is available free of charge to users of the Brazilian SUS. The ANVISA *bula* (i) highlights the increased risk of hypersensitivity reactions to abacavir in carriers of HLA‐B*57:01; (ii) indicates that testing for HLA‐B*57:01 “must be considered” (classified as *PGx test recommended*) prior to the first prescription of abacavir as well as prior to re‐exposure to abacavir, even in individuals who appeared tolerant to the drug; (iii) states that abacavir “is not recommended to patients with the HLA‐B*57:01 allele, or patients with suspected hypersensitivity reactions during pre‐exposure to abacavir‐containing drug regimens, irrespective of the HLA‐B*57:01 status”.

HLA‐B*57:01 carriers represent 3.6% of the REDOME cohort. Crovella et al.^14^ reported that three out of 96 HIV‐infected Brazilians (3.1%) treated with abacavir were HLA‐B*5701 carriers, and displayed hypersensitivity reactions characterized by cutaneous rash and gastrointestinal tract symptoms.

### 3.2 HLA‐B*15:02 and HLA‐A*31:01– carbamazepine, oxcarbazepine and phenytoin

#### 3.2.1 Carbamazepine

PGx testing for HLA‐B*15:02 is required by FDA and Swissmedic, and recommended by CHSC, while testing for HLA‐A*31:01 is recommended by CHSC and Swissmedic. The ANVISA *bula* (i) refers to “increasing evidence for the role of different HLA alleles in patients at risk for immuno‐mediated adverse reactions”, (ii) identifies HLA‐B*15:02 and HLA‐A*31:01 as risk alleles, (iii) describes the worlwide variability in prevalence of these alleles, and (iv) asserts that testing for the presence of HLA‐B*15:02 and HLA‐A*31:01 must be considered (classified as *PGx test recommended*) for patients genetically descendent from at‐risk populations, prior to initiating carbamazepine therapy.

#### 3.2.2 Oxcarbazepine

PGx testing for HLA‐B*15:02 is required by Swissmedic and recommended by FDA and CHSC, while testing for HLA‐A*31:01 is not required or recommended by the agencies listed in the PharmGKB Drug Label Annotations table. The ANVISA *bula* refers to the increased risk of cutaneous adverse reactions (CAR) to oxcarbazepine in carriers of HLA‐B*1502 or HLA‐A*31:01, and to the variable frequency of these alleles across populations worldwide. It is stated that testing for HLA‐B*1502 must be considered in patients with “descendance from populations at genetic risk prior to initiating treatment with oxcarbazepine”, but is not recommended in “populations in which the prevalence of the HLA‐B*15:02 allele is low, or patients already in use of oxicarbazepine”. With respect to HLA‐A*3101, the *bula* asserts that “there are no sufficient data to support recommendation for testing prior to initiating treatment with oxcarbazepine”.

#### 3.2.3 Phenytoin

The CHSC product monograph notes that *HLA‐B* genotyping should be considered as a screening tool in individuals of Asian ancestry and the use of phenytoin should be avoided in patients who test positive for the HLA‐B*15:02 allele. The ANVISA *bula* for phenytoin makes no reference to *HLA* haplotypes.

HLA‐B*15:02 is absent or extremely rare (0.1%) in Brazilians, whereas HLA‐A*31:01 was found in 9.3% of the REDOME cohort. A study of Brazilian patients who developed CAR during antiseizure medication reported absence of HLA‐B*15:02 carriers and no association of HLA‐A*31:01 with CAR in patients exposed to carbamazepine, oxcarbazepine or phenytoin.^15^

### 3.3 *CFTR*–ivacator

PGx testing is required by FDA, Swissmedic and CHSC. Brazil was the first Latin American country to make ivacaftor available in the public health system for cystic fibrosis patients older than 6 years having one of nine class III *CFTR* variants, or patients older than 18 years with an R117H causing variant. Accordingly, the ANVISA approved *bula* states that if the “CFTR genotype is unknown, an exact and validated genotyping method should be performed (classified as *PGx test required*) prior to initiating treatment, to confirm the presence of a (target) mutation in at least one *CFTR* allele”.

A study of 92 Brazilian cystic fibrosis patients revealed that only two (2.2%) fulfilled the ANVISA criteria for precription of ivacaftor; the majority of patients (76.1%) presented class I and II causing variants, the most prevalent being F508del, R1162X, G542X and N1303K.^16^

### 3.4 G6PD deficiency–pegloticase, primaquine, rasburicase and tafenoquine

PGx testing is required by FDA for the four drugs, by EMA for pegloticase and by CHSC and Swissmedic for rasburicase. Pegloticase is not marketed in Brazil. ANVISA *bulas* contraindicate primaquine and rasburicase for patients with G6PD deficiency and recommend testing for G6PD deficiency prior to initiating primaquine treatment for radical cure of *Plasmodium vivax* malaria; however, PGx testing is not required or recommended for rasburicase. Tafenoquine is approved by ANVISA for radical cure of *P. vivax* malaria in patients with G6PD activity >70% of the local population median; the *bula* asserts that “G6DP test should be performed (classified as *PGx test required*) prior to prescription of tafenoquine”. SUS incorporated G6PD phenotype testing to the tafenoquine treatment protocol for radical cure of malária.

Prevalence of G6PD deficiency was reported to range from 1.1 to 13.1% in distinct Brazilian cohorts^17^; in neonates from the three most populated regions of Brazil, the mean prevalence was reported at 4.75%.^18^

### 3.5 DPD phenotype–fluoropyrimidines

The Swissmedic drug label for fluorouracil requires *DPYD* testing in patients receiving fluorouracil who have also been treated with nucleoside analogues; FDA and CHSC recommend PGx testing prior to administration of fluorouracil. Regarding capecitabine, PGx testing is recommended by FDA, EMA and CHSC, but not Swissmedic. The fluorouracil *bula* makes no reference to PGx testing, while the capecitabine *bula* asserts that (i) “testing for DPD deficiency should be considered (classified as *PGx test recommended*), based on local availability and current guidelines” and (ii) patients with “complete or nearly complete loss of DPD activity” should not be treated with capecitabine.

DPD poor and intermediate metabolizers accounted, respectively, for 0.4% and 3.5% of 300 gastrointestinal cancer patients genotyped by our group at INCA for five *DPYD* polymorphisms (rs2918290 [DPYD*2A], rs55886062 [DPYD*13], rs67376798, rs56038477 (*HapB3*) and rs115232898).^19^ The absence of rs55886062 and the minor allele frequency (MAF) of the other SNPs in these patients is consistent with results from other Brazilian cohorts.^20^, ^21^

### 3.6 *NUDT15*–thiopurines (azathioprine, mercaptopurine and thioguanine)

The CHSC monograph for azathioprine indicates that genotypic analysis of *NUDT15* should be performed for all patients, including paediatric patients, prior to initiating thiopurine treatment; the FDA and Swissmedic labels describe PGx testing as recommended and actionable, respectively. For the two other thiopurines, PGx testing is recommended by FDA, EMA and CHSC for mercaptopurine, and by FDA and CHSC for thioguanine. The ANVISA *bulas* for the three thiopurines consistently alert to (i) lower tolerance and increased risk of adverse effects in carriers of *NUDT15* (and/or *TPMT*) reduced or no‐function alleles and (ii) significantly greater intolerance in carriers of variant alleles in both genes. There is an apparent discrepancy in the three *bulas* with respect to PGx testing for *NUDT15*: in section 3 (“Pharmacological characteristics”) it is stated that “genotypic analysis that determines *NUDT15* genotype *must be performed* for all patients, including pediatric patients”, whereas section 5 (“Warnings and Precautions”) asserts that “genotypic and phenotypic tests of *NUDT15* variants *must be considered* prior to starting therapy with thiopurines in all patients, including pediatric patients”. The alerts in sections 3 and 6 are classified by the author as *PGx testing required* and *PGx testing recommended*, respectively (Methods). For the comparative analysis with other drug regulatory agencies (see below), *PGx testing required* was selected for the three thiopurines.

In a cohort of paediatric acute lymphoblastic leukemia (ALL) patients (*n* = 430) from nine Brazilian cancer centers, genotyped by our group at INCA, the rs116855232 SNP which defines the no‐function NUDT15*2 and *3 alleles, occurred in heterozygosis with a MAF of 1.2%.^22^

### 3.7 *TPMT*–thiopurines (azathioprine, mercaptopurine and thioguanine)

*TPMT* genotyping is recommended by FDA for the three thiopurines, by CHSC for mercaptopurine and thioguanine and by EMA for mercaptopurine only. As mentioned above, the ANVISA *bulas* for the three drugs consistently refer to thiopurine intolerance in carriers of *TPMT* (and/or *NUDT15*) reduced or no‐function variants. However, the inserts for the three thiopurines differ with respect to PGx testing for *TPMT* risk variants: “the genotypic test may determine the allelic profile of a patient” (azathioprine); “some laboratories perform tests to detect TMPT deficiency … however, these tests do not identify all patients at risk for severe toxicity” (mercaptopurine); “it is recommended that the prescribing physician establish if a dose reduction is required, based on the patient response to treatment as well as on his/her genetic profile” (thioguanine). The PGx levels in the *bulas* for the three thiopurines were classified as *Actionable PGx*.

In the cohort of Brazilian paediatric ALL patients genotyped by our group (see above), the MAFs of *TPMT* variants were 4.9% (rs1142345), 2.4% (rs1800460) and 1.4% (rs1800462). The frequencies of the compound TPMT/NUDT15 metabolic phenotypes, associated with the CPIC dosing recommendations for thiopurines were 11.6% (intermediate metabolizer of either enzyme), 0.9% (TPMT poor metabolizers), 0.9%, (compound intermediate metabolizers); NUDT15 poor metabolizers were not detected and normal metabolizers for both enzymes accounted for 86.5% of the cohort.^22^

### 3.8 HLA‐B*58:01–allopurinol

PGx testing is recommended by FDA and CHSC and listed as actionable by Swissmedic. The allopurinol *bula* reads: (i) “HLA‐B*5801 has been identified as associated with risk of hypersensitivity and STS/TEN (Stevens‐Johnson syndrome/toxic epidermal necrolysis) caused by allopurinol”; (ii) “use of genotyping as a tool to make decisions about allopurinol treatment has not been established”; (iii) “if the patient is a known carrier of HLA‐B*58:01, use of allopurinol may be considered when benefits are superior to risks”. These annotations were classified as *Actionable PGx*.

HLA‐B*58:01 is present in 4.1% of the REDOME cohort. A study of 74 Brazilian patients with drug‐induced SCAR (severe cutaneeous adverse reactions) included 11 patients exposed to allopurinol, of whom five were heterozygous for HLA‐B*57:01, leading the authors to conclude that HLA genotyping allowed identification of risk alleles, thus “reinforcing their implication in SCAR induced by allopurinol and other drugs among the Brazilian population”.^23^

### 3.9 *SLCO1B1–*simvastatin

The Swissmedic label asserts that genotyping for the presence of the *SLCO1B1* c.521C allele “should be considered as part of the benefit–risk assessment in individual patients before prescribing simvastatin 80 mg, and high doses should be avoided in identified carriers of the CC genotype.” The ANVISA *bula* indicates that simvastatin is a substrate of “the transport protein OATP1B1” and that “co‐administration of inhibitors of OAT1B1 may lead to increased plasma concentration of simvastatin and increased risk of myopathy”, but no reference is made to PGx testing for *SLCO1B1* variants.

The MAF of the *SLCO1B1* c.521C in the overall Refargen cohort is 13.5%, with 2.5% carriers of the CC genotype.^24^ A study of hypercolesterolaemic Brazilian patients under treatment with simvastatin (20 mg) found no statistically significant influence of *SLCO1B1* haplotypes in the observed reductions in lipid and lipoprotein levels.^25^ A Pubmed search using the terms simvastatin AND *SLCO1B1* AND Brazil* disclosed no studies of association of *SLCO1B1* variants with simvastatin‐induced myopathy.

### 3.10 Distribution of the PGx annotations in the ANVISA *bulas*

**FIGURE 2:**
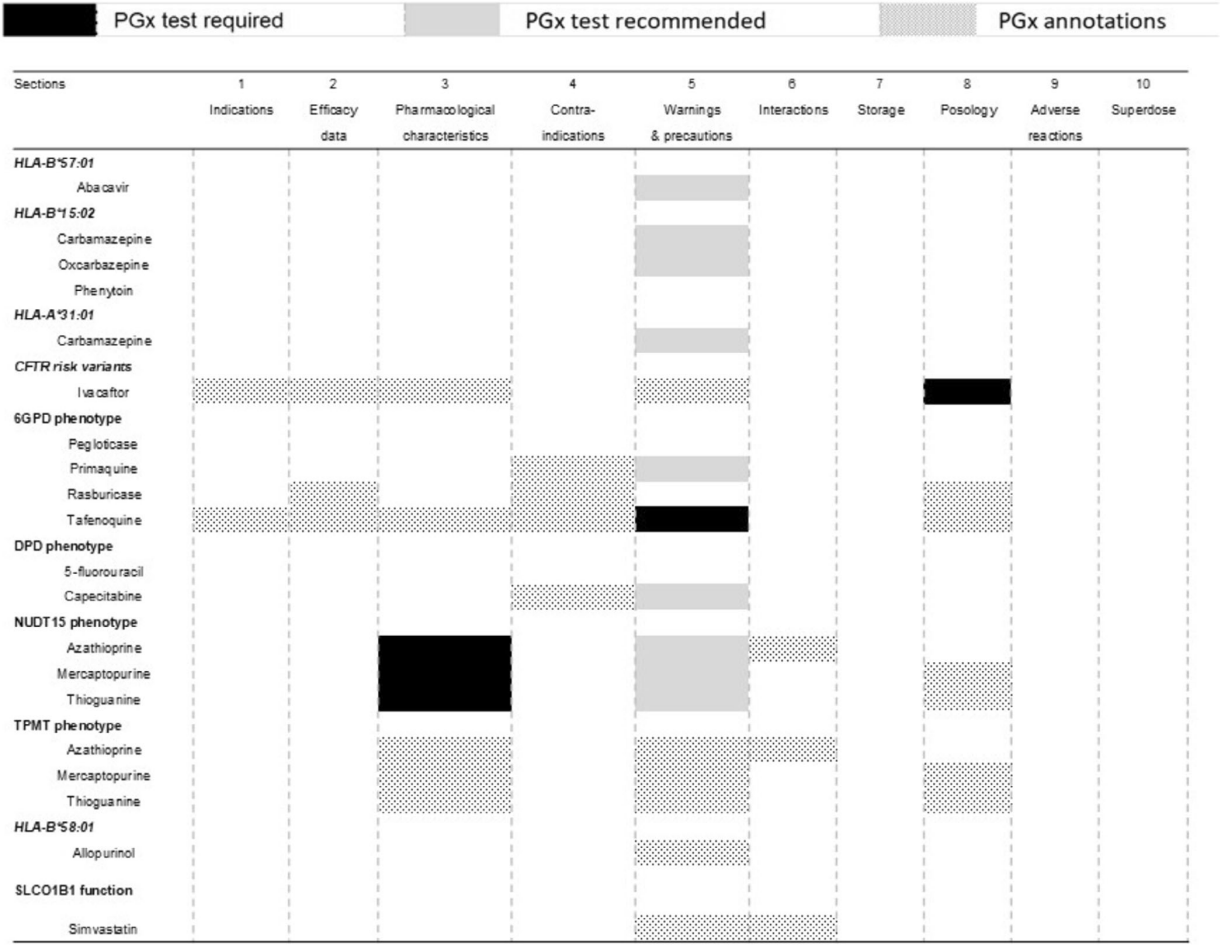
Distribution of PGx annotations across sections of the ANVISA *bulas*.

Collectively, the PGx annotations in ANVISA *bulas* analysed in this study were classified as *PGx testing required* (*n* = 5), *PGx testing recommended* (*n* = 6) and *Actionable PGx* (*n* = 4); there is no reference to PGx testing and/or PGx biomarkers in 4 *bulas*, and pegloticase is not marketed in Brazil. The PGx annotations are distributed among seven of the ten sections of ANVISA *bulas* (Figure 2): Section 5, denoted Warnings and Precautions (*Advertências e Precauções* in Brazilian Portuguese) shows PGx annotations in 16 of the 19 *bulas* (84.2%), including all annotations classified as *PGx testing recommended* and one classified as *PGx testing required* (tafenoquine‐G6PD defficiency); other annotations classified as “PGx testing required” appear in sections 3 (Pharmacological characteristics, *n* = 3) and 8 (Posology, *n* = 1). As mentioned above, the thiopurine *bulas* led to conflicting classification of PGx levels in sections 3 and 5.

### 3.11 Concordance in PGx annotations among regulatory agencies

**FIGURE 3:**
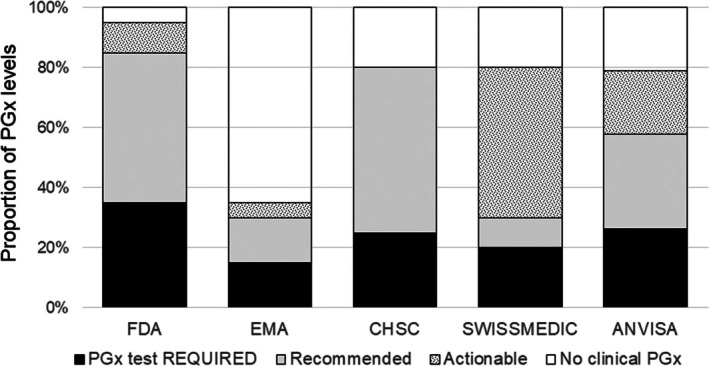
Distribution of assigned PGx levels in the PharmGKB Drug Label Annotation table (https://www.pharmgkb.org/labelAnnotations) and PGx levels classified by the author from the ANVISA *bulas*. FDA (Food and Drug Administration, USA), EMA (European medicines agency, European Union), CHSC (Health Canada Santé Canada), Swissmedic (Swiss Agency for Therapeutic Products), ANVISA (Brazilian Health Regulatory Agency).

Pairwise comparisons of the author‐classified PGx levels in ANVISA *bulas vs*. the assigned levels for EMA, FDA, HCSC and Swissmedic in the PharmGKB Drug Label Annotations table disclosed “poor” concordance (Cohen's kappa coefficient, *κ* < 0.20) for all pairs (Figure 3); the kappa value for ANVISA *vs*. CHS, *κ* < 0.19, approached but did not reach “fair” agreement. Poor concordance among assigned PGx levels by EMA, FDA, HCSC and Swissmedic for the 20 gene–drug pairs was also verified, with Fleiss's kappa coefficient *κ* = 0.08. Indeed, only for HLA‐B*57:01–abacavir are the four agencies in complete agreement, with PGx testing being required; FDA, EMA and CHSC (but not Swissmedic) agreed on assigned PGx levels to *CFTR–*ivacaftor (PGx testing required), DPD–capecitabine, *TPMT–*mercaptopurine and *NUDT15*–mercaptopurine (PGx testing recommended).

## 4 DISCUSSION

Adoption of PGx‐guided prescription in Brazil has been sporadic, as a likely result of a combination of factors, some specific to Brazil and the structure of the Brazilian population, and others that are recognized as barriers to implementation of PGx‐informed prescription worldwide.^26^ Of note, commercially available PGx tests are not reimbursed by the Brazilian SUS. At the present time, SUS requires and provides PGx tests for *CFTR*‐ivacaftor, G6PD‐tafenoquine and HLA‐B*57:01‐abacavir. The three gene‐drug pairs fulfil the inclusion criteria of the present analysis, namely CPIC strong or moderate recommendations for initial dosing alteration and PGx test required or recommended by at least one of the regulatory agencies in the PharmGKB Drug Label Annotations table. Accordingly, the ANVISA‐approved *bulas* assert (i) “if the (CFTR) genotype is unknown, an exact and validated genoyping method should be performed prior to initiate treatment with ivacaftor” and (ii) “due to the risk of haemolysis in patients with G6PD deficiency or unknown G6PD status”, G6PD testing must be performed prior to prescription of tafenquine; the author classified both assertives as “*PGx testing required*”. The abacavir *bula*, however, informs that testing for HLA‐B*57:01 “must be considered”, which is classified as “*PGx testing recommended*”; this apparent discordance with the SUS requirement of PGx testing for HLA‐B*57:01 prior to prescription of abacavir highlights the need for reviewing the content of the ANVISA *bulas* to ascertain concordance with SUS determinations.

The ANVISA *bulas* are organized in 10 different sections with defined headings, but no section is dedicated to PGx. The *bulas* examined in this study have PGx annotations distributed over seven sections, with most annotations classified as *PGx testing recommended* or *PGx testing required*, listed in section 5, denoted Warnings and Precautions. The author suggests that addition of a PGx section to the *bulas*, with consistent phrasing regarding testing requirements, would avoid dispersion of clinically relevant PGx information across sections, assure integration of this information with SUS determinations and prevent discrepancies across sections, as observed in the *bulas* for the thiopurines, which might confuse healthcare providers. A similar proposal has been advanced for drug labels approved by the Italian Drug Agency (AIFA), and a prototype illustrative section was presented.^11^

Of the five drug–gene pairs classified as *PGx testing required* by ANVISA, three (*CFTR–*ivacaftor, *G6PD*–rasburicase and *NUDT15–*azathioprine) are also assigned the same PGx level by at least one of the regulatory agencies in the PharmGKB Drug Label Annotations table, while the other two pairs (*NUDT15–*mercaptopurine and *NUDT15–*thioguanine) have PGx testing recommended by these agencies. Of the six gene–drug pairs classified as *PGx testing recommended* by ANVISA, 4 (HLA‐A*31:01–carbamazepine, HLA‐B*15:02–carbamazepine, HLA‐B*15:02–oxcarbazepine and *DPYD*–capecitabine) are assigned *PGx testing recommended* by at least one of the regulatory agencies, and 2 (HLA‐B*57:01–abacavir and *G6PD–*primaquine) have *PGx testing required* by these agencies. Pairwise comparison of the PGx levels in ANVISA *bulas vs*. FDA, EMA, CHSC and Swissmedic revealed poor concordance, with Cohen's kappa coefficient *κ* < 0.20 for all pairs. For comparison, the PGx levels assigned by AIFA, the Italian Drug Agency, for 311 drug–gene pairs were in “fair agreement with EMA (*κ* = 0.341) and FDA (*κ* = 0.267), while agreement with HCSC (*κ* = 0.260), PMDA (κ = 0.169) and Swissmedic (*κ* = 0.105) was minimal”.^11^ Of note, discordance among assigned PGx levels by FDA, EMA, CHSC, Swissmedic and PMDA is also considerable: the Fleiss kappa statistics pointed to poor agreement for the 20 gene–drug pairs of our study (*κ* = 0.08), while 36% discordant classifications were reported for the 311 gene–drug pairs examined by Moschella et al.^11^

Although agreement of assigned PGx levels across regulatory agencies may prove advantageous to achieve consensual international PGx recommendations, some discordance is to be expected and justified, as a consequence of the impact of population diversity on the worldwide distribution of clinically relevant PGx biomarkers. For example, the high‐risk HLA‐B*15:02 allele associated with carbamazepine‐ and oxcarbazepine‐induced SCAR, is extremely rare in Brazilians (MAF 0.03% in REDOME) but common (MAF > 10%) in several Asian populations. The ANVISA *bula* asserts that genotyping *HLA‐B* must be considered in patients with “descendance from populations at genetic risk prior to initiating treatment with oxcarbazepine”, but is not recommended in “populations in which the prevalence of the HLA‐B*1502 allele is low”. Accordingly, the FDA‐approved label states “testing for HLA‐B*1502 should be performed in patients with ancestry in populations in which HLA‐B*1502 may be present”, prior to initiating carbamazepine therapy. Nonetheless, population diversity does not solely account for, or is the major determinant of, the discordance in PGx levels among regulatory agencies.

Previous studies have shown that 93.5% of Brazilians have actionable genotypes in five *CYP* clinically relevant pharmacogenes^27^ and that 98.0% “carry at least one high risk genotype‐predicted phenotype in pharmacogenes with PharmGKB level of evidence 1A for drug interaction”.^21^ These findings are consistent with the notion that the vast majority of individuals from distinct populations worldwide have at least one clinicaly actionable PGx variant (Introduction). However, the frequency of the PGx risk biomarkers for the 20 gene–drug pairs selected for this study range from <0.1% (*CFTR* risk variants and HLA‐B*15:02 carriers) to 10.8% (TPMT IMs plus PMs), adding to 42.5% in representative cohorts of the overall Brazilian population (Table 1). The actual combined prevalence is likely lower—since individuals may carry more than one biomarker—and may vary across race/Colour groups, due to their different average proportions of Native American, European and subSaharan Africa ancestry.^28^ Indeed, carriers of *HLA* risk alleles range in frequency from 7.9–9.9% (HLA‐A*31:01), 2.6–4.2% (HLA‐B*57:01) and 3.6–5.2% (HLA‐B*58:01) across self‐reported White, Brown and Black individuals of the REDOME cohort; the differences among these race/Colour groups although statistically significant (chi square *P* < 0.001), do not exceed 2% for any of these *HLA* risk variants. Similar differences in frequency of other biomarkers, such as the *SCLO1B1* c.521C/C genotype^23^ and *TPMT* phenotypes^29^ across self‐identified White, Brown and Black cohorts have been reported, but it is the author's contention that selection of variants for PGx testing panels and implementation of PGx‐informed dosing guidelines for Brazilians should not be influenced by race/Colour categories.^30^ Nevertheless, it must be emphasized that Native Americans, who account for 0.4% of the current Brazilian population, distributed over >300 ethnic groups and speaking almost 300 languages, have been reported to display wide PGx diversity, both interethnically and in relation to non‐Indigenous Brazilians, Two biomarkers from the present study provide remarkable examples: (i) *NUDT15* rs116855232 ranges in frequency from 1.7% in Yanomami from the Brazilian Amazon^31^ to 31.7% Kaingang from Brazil's South region.^32^ compared to 1.2% in non‐Indigeneous ALL patients genotyped at INCA^22^; (ii) TPMT*3A haplotype ranges in frequency from 0.6% in Yanomami to 18.8% of Paiter‐Surui from the Brazilian Amazon,^31^ compared to 2.1% in the non‐Indigenous ALL patients.^22^

The following limitations of this study are recognized: first, the ANVISA *bulas* do not explicitly provide PGx levels according to PharmGKB, and PGx levels were classified by the author based on the *bulas*' text. Although this is a potential source of subjectivity, the wording in the *bulas* which led to PGx testing being classified by the author as *recommended* or *required* was clearly distinct: PGx testing was classified as recommended when the Portuguese verb *considerar* (to consider) was used as in the abacavir bula, and classified as required when the verbs *realizar* or *determinar* (to carry out, to determine) were used as in the thioguanine and ivacaftor bulas, respectively. Second, the criteria adopted for inclusion of gene–drug pairs in this study led to exclusion of (i) PGx testing for tumour somatic variants, as these are not covered by published CPIC guidelines; (ii) biomarker–drug pairs with CPIC strong or moderate recommendation for initiating treatment with standard dosing (e.g., CYP2D6 metabolic phenotypes–metoprolol); (iii) gene–drug pairs with CPIC strong or moderate recommendations for alteration of initial dosing but no PGx testing required or recommended by regulatory agencies in the PharmGKB Drug Label Annotation table (e.g. *CYP3A5–*tacrolimus); (iv) conversely, gene–drug pairs with PGx testing required or recommended by these agencies and no CPIC guideline: *UGT1A1–*irinotecan provides a remarkable example, since PGx testing is recommended by FDA, HCSC and PMDA, while the DPWG guideline for irinotecan considers *UGT1A1* genotyping “essential—indicating that *UGT1A1* testing must be performed prior to initiating irinotecan treatment”, and a 70% starting dose in UGT1A1 poor metabolizers is recommended^33^; however, no CPIC guideline for irinotecan has yet been published. The ANVISA *bula* for irinotecan asserts that *UGT1A1* genotyping “may be useful to identify patients at rish for adverse effects”, which the author classifies as “*Actionable PGx*”. The frequency of the high‐risk UGT1A1 PM phenotype in non‐Indigenous Brazilians has been reported to range between 10 and 18%,^28^, ^34^, ^35^ compared to 18.8% in Paiter‐Suruí and 20.5% in Yanomami cohorts.^36^

## 5 CONCLUSIONS

PGx information in drug package inserts corroborate with evidence‐based guidelines to facilitate the translation of PGx findings to prescribing decisions in clinical practice. PGx annotations are distributed among several sections of the ANVISA‐approved *bulas* examined in this article, and were classified as PGx testing required (*n* = 5), PGx testing recommended (*n* = 5) and Actionable PGx (*n* = 4); four *bulas* had no PGx clinical information (*n* = 4). The PGx levels in several ANVISA *bulas* differed from those assigned by international regulatory agencies; however, discordance among these agencies is also considerable. Population diversity may justify distinct PGx testing requirements worldwide, but does not solely account for the prevailing discordance among regulatory agencies.

### 5.1 Nomenclature of targets and ligands

Key protein targets and ligands in this article are hyperlinked to corresponding entries in http://www.guidetopharmacology.org, and are permanently archived in the Concise Guide to PHARMACOLOGY 2021/22.^37^

## AUTHOR CONTRIBUTIONS

G.S‐K is the sole author of this review.

## CONFLICT OF INTEREST STATEMENT

There are no conflicts of interest to declare.

## Supporting information


**Table S1** Links to CPIC guidelines (last accessed May 25, 2025)
**Table S2** Links to the ANVISA‐approved package inserts (*bulas*)*.
**Figure S1**. CPIC dosing recommendatins according to PGx biomarker.

## Data Availability

All data presented are published in the referenced sources.
