# Supplementary material for: Pharmacogenomics in clinical practice: Biomarker information in Brazilian drug labels
Source: Br J Clin Pharmacol. 2025 Sep 28;92(1):109–18. doi: 10.1002/bcp.70278 (PMC12746351; doi:10.1002/bcp.70278)
Supplement: Supplementary file 1 — Table S1 Links to CPIC guidelines (last accessed May 25, 2025) Table S2 Links to the ANVISA‐approved package inserts (bulas)*. Figure S1. CPIC dosing recommendatins according to PGx biomarker. [file BCP-92-109-s001.docx]

**Supplementary File 1. Links to CPIC guidelines (last accessed May 25, 2025)**

| https://cpicpgx.org/guidelines/guideline-for-ivacaftor-and-cftr/ |
| --- |
| https://cpicpgx.org/guidelines/cpic-guideline-for-efavirenz-based-on-cyp2b6-genotype/ |
| https://cpicpgx.org/guidelines/cpic-guideline-for-methadone-based-on-cyp2b6-genotype/ |
| https://cpicpgx.org/guidelines/guideline-for-clopidogrel-and-cyp2c19/ |
| https://cpicpgx.org/guidelines/cpic-guideline-for-proton-pump-inhibitors-and-cyp2c19/ |
| https://files.cpicpgx.org/data/guideline/publication/voriconazole/2016/27981572.pdf |
| https://cpicpgx.org/guidelines/cpic-guideline-for-nsaids-based-on-cyp2c9-genotype/ |
| https://cpicpgx.org/guidelines/guideline-for-phenytoin-and-cyp2c9-and-hla-b/ |
| https://cpicpgx.org/guidelines/guideline-for-warfarin-and-cyp2c9-and-vkorc1/ |
| https://cpicpgx.org/guidelines/cpic-guideline-for-atomoxetine-based-on-cyp2d6-genotype/ |
| https://cpicpgx.org/guidelines/guideline-for-ondansetron-and-tropisetron-and-cyp2d6-genotype/ |
| https://cpicpgx.org/guidelines/cpic-guideline-for-tamoxifen-based-on-cyp2d6-genotype/ |
| https://cpicpgx.org/guidelines/guideline-for-tricyclic-antidepressants-and-cyp2d6-and-cyp2c19/ |
| https://cpicpgx.org/guidelines/cpic-guideline-for-ssri-and-snri-antidepressants/ |
| https://cpicpgx.org/guidelines/guideline-for-codeine-and-cyp2d6/ |
| https://cpicpgx.org/guidelines/guideline-for-tacrolimus-and-cyp3a5/ |
| https://cpicpgx.org/guidelines/guideline-for-fluoropyrimidines-and-dpyd/ |
| https://cpicpgx.org/guidelines/cpic-guideline-for-g6pd/ |
| https://cpicpgx.org/guidelines/guideline-for-carbamazepine-and-hla-b/ |
| https://cpicpgx.org/guidelines/guideline-for-abacavir-and-hla-b/ |
| https://cpicpgx.org/guidelines/guideline-for-allopurinol-and-hla-b/ |
| https://cpicpgx.org/guidelines/guideline-for-peg-interferon-alpha-based-regimens-and-ifnl3/ |
| https://cpicpgx.org/guidelines/guideline-for-peg-interferon-alpha-based-regimens-and-ifnl3/ |
| https://cpicpgx.org/guidelines/cpic-guideline-for-aminoglycosides-and-mt-rnr1/ |
| https://cpicpgx.org/guidelines/cpic-guideline-for-ryr1-and-cacna1s/ |
| https://cpicpgx.org/guidelines/cpic-guideline-for-statins/ |
| https://cpicpgx.org/guidelines/guideline-for-thiopurines-and-tpmt/ |
| https://cpicpgx.org/guidelines/guideline-for-atazanavir-and-ugt1a1/ |

| **Supplementary Table 2 Links to the ANVISA-approved package inserts (*bulas*)*** | |
| --- | --- |
| **Drug** | **Links to ANVISA *bulas*** |
| Abacavir | https://consultas.anvisa.gov.br/#/bulario/q/?nomeProduto=ZIAGENAVIR |
| Allopurinol | https://consultas.anvisa.gov.br/#/bulario/q/?nomeProduto=ALOPURINOL |
| Azathioprine | https://consultas.anvisa.gov.br/#/bulario/q/?nomeProduto=IMURAN |
| Capecitabine | https://consultas.anvisa.gov.br/#/bulario/q/?nomeProduto=CAPECITABINA |
| Carbamazepine | https://consultas.anvisa.gov.br/#/bulario/q/?nomeProduto=CARBAMAZEPINA |
| Fluorouracil | https://consultas.anvisa.gov.br/#/bulario/q/?nomeProduto=FLUORURACILA |
| Irinotecan | https://consultas.anvisa.gov.br/#/bulario/q/?nomeProduto=CAMPTOSAR |
| Ivacaftor | https://consultas.anvisa.gov.br/#/bulario/q/?nomeProduto=KALYDECO |
| Mercaptopurine | https://consultas.anvisa.gov.br/#/bulario/q/?nomeProduto=PURINETHOL |
| Oxcarbazepine | https://consultas.anvisa.gov.br/#/bulario/q/?nomeProduto=OXCARBAZEPINA |
| Phenytoin | https://consultas.anvisa.gov.br/#/bulario/q/?nomeProduto=FURP%20-%20FENITOINA |
| Primaquine | https://consultas.anvisa.gov.br/#/bulario/q/?nomeProduto=FARMANGUINHOS%20PRIMAQUINA |
| Rasburicase | https://consultas.anvisa.gov.br/#/bulario/q/?nomeProduto=FASTURTEC |
| Simvastatine | https://consultas.anvisa.gov.br/#/bulario/q/?nomeProduto=SINVASTATINA |
| Tafenoquine | https://consultas.anvisa.gov.br/#/bulario/q/?nomeProduto=Kozenis |
| Thioguanine | https://consultas.anvisa.gov.br/#/bulario/q/?nomeProduto=LANVIS |

* last accessed May 25, 2025

|  | | | | | | | | | |
| --- | --- | --- | --- | --- | --- | --- | --- | --- | --- |
|  | | | | | | | | | |
| **Supplementary Figure 1. CPIC dosing recommendatins according to PGx biomarker** | | | | | | | | | |
| **Color code** | | **CPIC Level** | | | **Dosing recommendation** | | |  | |
|  | STRONG | | | Alteration of initial dose | | | | |  |
|  | STRONG | | | Use standard initial dose | | | | |  |
|  | Moderate | | | Alteration of initial dose | | | | |  |
|  | Moderate | | | Use standard initial dose | | | | |  |
|  | Optional | | | Standard or alteration of initial dose | | | | |  |
|  | |  |  | |  |  |  |  | |
| **Gene/drug pairs** | | **PGx biomarker (genotype or phenotype)** | | | | | | |  |
| **CYP3A5 phenotype** | | **NM** | **IM** | | **PM** |  |  |  | |
| Tacrolimus | |  |  | |  |  |  |  | |
|  | |  |  | |  |  |  |  | |
| **CYP2B6 phenotype** | | **NM** | **IM** | | **PM** | **RM** | **UM** |  | |
| Efavirenz | |  |  | |  |  |  |  | |
| Methadone | |  |  | |  |  |  |  | |
| Sertraline | |  |  | |  |  |  |  | |
|  | |  |  | |  |  |  |  | |
| **CYP2C19 phenotype** | | **NM** | **IM** | | **PM** | **RM** | **UM** |  | |
| Voriconazole | |  |  | |  |  |  |  | |
| Clopidodrel (ACS and/or PCI) | |  |  | |  |  |  |  | |
| Clopidodrel (non ACS, non PGI) | |  |  | |  |  |  |  | |
| Clopidodrel (non CV indications) | |  |  | |  |  |  |  | |
| Amitriptyline, nortripyline | |  |  | |  |  |  |  | |
| Other TCAs | |  |  | |  |  |  |  | |
| Omeprazole, esomeprazole | |  |  | |  |  |  |  | |
| Lansoprazole | |  |  | |  |  |  |  | |
| Pantoprazole | |  |  | |  |  |  |  | |
| Dexlansoprazole | |  |  | |  |  |  |  | |
| Citalopram | |  |  | |  |  |  |  | |
| Escitalopram | |  |  | |  |  |  |  | |
| Sertraline | |  |  | |  |  |  |  | |
| **CYP2C9 phenotype** | | **NM** | **IM AS 1.5** | | **IM AS 1.0** | **PM** |  |  | |
| Celecoxib | |  |  | |  |  |  |  | |
| Flurbiprofen | |  |  | |  |  |  |  | |
| Lornoxicam, ibuprofen | |  |  | |  |  |  |  | |
| Meloxicam | |  |  | |  |  |  |  | |
| Piroxicam | |  |  | |  |  |  |  | |
| Tenoxicam | |  |  | |  |  |  |  | |
| Fluvastatin | |  |  | |  |  |  |  | |
| Phenytoin (HLA-B*15:02 negative) | |  |  | |  |  |  |  | |
| Warfarin - non-African ancestry | |  |  | |  |  |  |  | |
| Warfarin - African ancestry | |  |  | |  |  |  |  | |
|  | |  |  | |  |  |  |  | |
| **CYP2D6 phenotype** | | **NM** | **IM** | | **PM** | **UM** |  |  | |
| Ondansetron, tropisetron | |  |  | |  |  |  |  | |
| Amitriptyline, nortriptyline | |  |  | |  |  |  |  | |
| Other TCAs | |  |  | |  |  |  |  | |
| Tamoxifen | |  |  | |  |  |  |  | |
| Atomoxetine (adults) | |  |  | |  |  |  |  | |
| Atomoxetine (children) | |  |  | |  |  |  |  | |
| Tramadol | |  |  | |  |  |  |  | |
| Codeine | |  |  | |  |  |  |  | |
| Hydrocodone | |  |  | |  |  |  |  | |
| Paroxetine | |  |  | |  |  |  |  | |
| Fluvoxamine | |  |  | |  |  |  |  | |
| Venlafaxine | |  |  | |  |  |  |  | |
| Vortioxetine | |  |  | |  |  |  |  | |
| Metoprolol | |  |  | |  |  |  |  | |
| **DPD phenotype** | | **NM** | **IM AS 1.5** | | **IM AS 1.0** | **PM** |  |  | |
| Capecitabine | |  |  | |  |  |  |  | |
| 5-fluorouracil | |  |  | |  |  |  |  | |
| **UGT1A1 phenotype** | | **NM** | **IM** | | **PM** |  |  |  | |
| Atazanavir | |  |  | |  |  |  |  | |
| **SLCO1B1 function** | | **Normal** | **Decreased** | | **Poor** | **Increased** |  |  | |
| Atorvastatin, Fluvavstatin | |  |  | |  |  |  |  | |
| Lovastatin | |  |  | |  |  |  |  | |
| Pitavastatin | |  |  | |  |  |  |  | |
| Pravastatin | |  |  | |  |  |  |  | |
| Rosuvastatin | |  |  | |  |  |  |  | |
| Simvastatin | |  |  | |  |  |  |  | |
|  | |  |  | |  |  |  |  | |
| **ABCG2 phenotype (function)** | | **Normal** | **Decreased** | | **Poor** |  |  |  | |
| Rosuvastatin | |  |  | |  |  |  |  | |
|  | |  |  | |  |  |  |  | |
| **TPMT phenotype** | | **NM** | **IM** | | **PM** |  |  |  | |
| Azathioprine | |  |  | |  |  |  |  | |
| Mercaptopurine | |  |  | |  |  |  |  | |
| Thioguanine | |  |  | |  |  |  |  | |
|  | |  |  | |  |  |  |  | |
| **NUDT15 phenotype** | | **NM** | **IM** | | **PM** |  |  |  | |
| Azathioprine | |  |  | |  |  |  |  | |
| Mercaptopurine | |  |  | |  |  |  |  | |
| Thioguanine | |  |  | |  |  |  |  | |
|  | |  |  | |  |  |  |  | |
| **CFTR risk variants** | | **Non Carrier** | **Carrier** | |  |  |  |  | |
| Ivacaftor | |  |  | |  |  |  |  | |
|  | |  |  | |  |  |  |  | |
| **HLA-A*31:01** | | **Negative** | **Positive** | |  |  |  |  | |
| Carbamazepine | |  |  | |  |  |  |  | |
|  | |  |  | |  |  |  |  | |
| **HLA-B*15:02** | | **Negative** | **Positive** | |  |  |  |  | |
| Carbamazepine | |  |  | |  |  |  |  | |
| Oxcarbazepine | |  |  | |  |  |  |  | |
| Phenytoin | |  |  | |  |  |  |  | |
| **HLA-B*57:01** | | **Noncarrier** | **Carrier** | |  |  |  |  | |
| Abacavir | |  |  | |  |  |  |  | |
|  | |  |  | |  |  |  |  | |
| **HLA-B*58:01** | | **Noncarrier** | **Carrier** | |  |  |  |  | |
| Allopurinol | |  |  | |  |  |  |  | |
|  | |  |  | |  |  |  |  | |
| **6GPD phenotype** | | **Normal** | **Deficient** | |  |  |  |  | |
| Pegloticase | |  |  | |  |  |  |  | |
| Primaquine | |  |  | |  |  |  |  | |
| Rasburicase | |  |  | |  |  |  |  | |
| Tafenoquine | |  |  | |  |  |  |  | |
|  | |  |  | |  |  |  |  | |
| ***INFL3* genotype** | | **Favorable** | **Unfavorable** | |  |  |  |  | |
| PEG-IFN-alfa and RBV regimens | |  |  | |  |  |  |  | |
|  | |  |  | |  |  |  |  | |
| **MT-RNR1 phenotype** | | **Normal**  **risk** | **Increased risk** | |  |  |  |  | |
| Aminoglycosides (gentamycin) | |  |  | |  |  |  |  | |
|  | |  |  | |  |  |  |  | |
| ***RyR1/CCNA1S*** | | **Uncertain** | **MHS** | |  |  |  |  | |
| Volatile anesthetics (desflurane) | |  |  | |  |  |  |  | |
| Succinylcholyne | |  |  | |  |  |  |  | |
|  | |  |  | |  |  |  |  | |
| ***VKORC1* -1939G>A** | | **GG** | **GA** | | **AA** |  |  |  | |
| Warfarin - non-African ancestry | |  |  | |  |  |  |  | |
| Warfarin - African ancestry | |  |  | |  |  |  |  | |
